# Elaboration and Characterization of Different Zirconium Modified ETS Photocatalysts for the Degradation of Crystal Violet and Methylene Blue

**DOI:** 10.1002/open.202400348

**Published:** 2024-11-13

**Authors:** Hristina I. Lazarova, Rusi I. Rusew, Liliya V. Tsvetanova, Borislav Z. Barbov, Elena S. Tacheva, Boris L. Shivachev

**Affiliations:** ^1^ Institute of Mineralogy and Crystallography “Acad. Ivan Kostov” – Bulgarian Academy of Sciences (IMC-BAS) Acad. G. Bonchev Str., Bl. 107 1113 Sofia Bulgaria

**Keywords:** Photodegradation, Zr, Engelhard titanosilicate, Methylene blue, crystal violet

## Abstract

In this study, Zirconium‐modified Engelhard Titanium Silicate 4 (Na−K‐ETS‐4/xZr) catalysts were synthesized and evaluated for their photocatalytic efficiency in degrading crystal violet (CV) and methylene blue (MB) in aqueous solutions. The catalysts were characterized using XRD, FTIR, SEM, WDXRF, and nitrogen adsorption/desorption isotherms. The results confirmed the successful incorporation of Zr into the ETS‐4 framework, with the highest Zr content reaching 9.2 wt %. The photocatalytic performance under visible light irradiation was studied at varying pH levels. The Na−K‐ETS‐4/6.3Zr catalyst exhibited the highest photodegradation efficiency for CV (76.6 %), while Na−K‐ETS‐4/8.9Zr achieved 86.6 % efficiency for MB. A combination of Engelhard Titanium Silicate 10, Na−K‐ETS‐10/6.3Zr and Na−K‐ETS‐4/8.9Zr significantly enhanced dye degradation, achieving up to 96.5 % efficiency for MB. Kinetic studies indicated that the degradation process follows a non‐linear pseudo‐first‐order model. The catalysts also demonstrated excellent reusability, with minimal efficiency loss after five cycles, and full recovery after an ethanol wash. These findings suggest that Na−K‐ETS‐4/xZr is a promising candidate for environmental water treatment applications due to its efficient photodegradation performance and stability.

## Graphical abstract

The study emphasizes ETS‐4′s potential for environmental applications, showcasing its ability to purify water from industrial dyes through a sustainable, reusable and rapid process in just 30 minutes per cycle. Utilizing sunlight, Zr modified ETS‐4 efficiently promotes photodegradation of crystal violet and methylene blue, maintaining over 90 % effectiveness across multiple cycles.

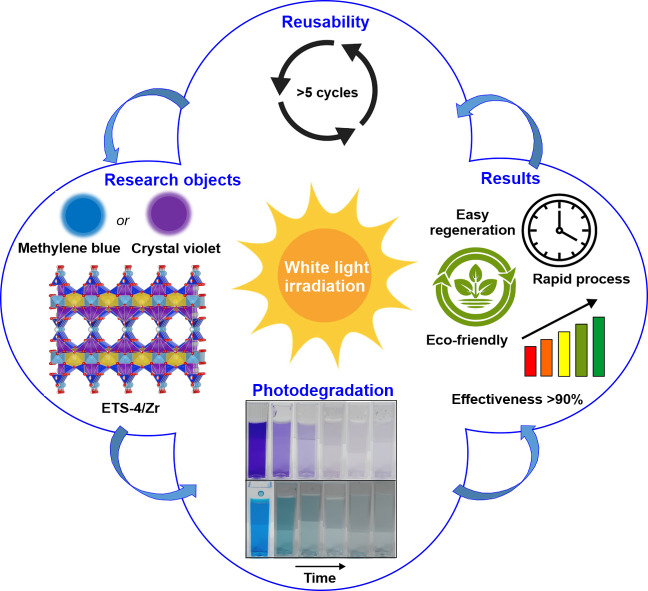

## Introduction

The dyes application finds a significant role in various sectors, including textiles, paper, cosmetics, food and pharmaceuticals industries.^[1]^ The textile industry accounts for ~75 % of the global dyestuff market and involves around ten thousand different dyes used for printing and/or coloring multiple types of fabrics.^[2]^ Initially, dyes were exclusively sourced from natural materials. However, with the rise in global population, the demand for dyes shifted towards synthetic alternatives. The research and industry trend was to produce cost‐effective, stable and unchanging dyes.^[3]^ In this regard, three main classes of dyes – cationic, anionic and non‐ionic – differing in the ionic charge carried by their structure have emerged. Although, great progress was made in dyestuff development, the industry faced substantial challenges, many of which pose major ecological and health risks. For example, the dyes production is an energy and water‐intensive process, leading to significant resource depletion. An additional drawback is that produced synthetic dyes may involve toxic and non‐biodegradable moieties that can lead to significant aquatic toxicity and subsequent soil pollution. Cationic dyes in particular are found to be more toxic than the other classes.^[4]^ Representatives such as crystal violet (CV) and methylene blue (MB) can cause various diseases, such as breathing disorders, eye irritation, vomiting, increased heart rate, burning skin, cancer, shock and convulsions, cyanosis, mutagenicity, jaundice, blindness and eye irritation.^[5]^ Embracing green chemistry and sustainability within dyestuff industry is crucial to mitigate these problems and thus nowadays the focus is shifting towards biodegradation, biodegradable dyes and environmental safety.

To address this issue, i. e. the removal of dyes from industrial wastewater, several methods have recently been proposed, such as photodegradation, adsorption, chemical coagulation, oxidation and membrane filtration.^[6]^ From those methods, photodegradation is considered as one of the most competitive technologies due to its cost‐effectiveness, high efficiency and simplicity. One of the difficulties accompanying the photodegradation process is the need of ultraviolet energy due to the pronounced stability of dyestuffs towards light/oxidants. In order to avoid the use of UV light in the photodegradation process, novel photoactive catalysts are required. Various materials have been investigated for this purpose, including ZnS,^[7]^ TiO_2_,^[8]^ ZnO,^[9]^ hematite,^[10]^ plasmonic metals (gold, silver and platinum),^[11]^ Ag_2_S@TiO_2_ nanofibers^[12]^ metal vanadates (those of Bi, Ni, Cu, Zn etc.),^[13]^ carbon‐based catalysts such as graphene and its oxides, and carbon nitrides,^[14]^ magnetite nanoparticles (NPs) and iron (III) oxide‐based catalysts.^[15]^

Other suitable candidates for photocatalytic applications are the zeolites, also known as molecular sieves or synthetic‐like zeolites. They are aluminosilicate minerals composed of a three‐dimensional framework of SiO_4_ and AlO_4_ tetrahedra linked by shared oxygen atoms, forming a regular, microporous structure with uniform pore sizes. Several hundred natural zeolite compounds are known^[16]^ combining well‐defined structures, high specific surface areas, shape selective pore systems, confinement effects and environmental tolerance. These properties are of great interest to researchers working in the field of photocatalysis due to the presence of a strictly defined surface that consists of a crystalline structure and close‐sized pores and cavities. A basic prerequisite for their use in photocatalytic processes is ensuring high selectivity with respect to the size and shape of reactants, intermediates and products, which makes zeolite materials preferred catalysts.^[17]^ One could say that the chemical composition of zeolite and synthetic‐like zeolites materials is shaping their acid‐base character and thermal stability, the framework topology determines the size and connectivity of channels and cavities and finally the incorporation of heteroelements/atoms affects the adsorption and catalytic activity of the sites. Substituting metals in synthetic‐like zeolites plays a crucial role in developing innovative catalytic processes that align with the principles of green chemistry and promote sustainability in the chemical industry and other areas of daily life. The advantages of metal‐substituted molecular sieves include high surface areas, molecular sieving effects, confinement effects, morphology, variability and stability in active sites. Important example for such metal substitution in zeolites are the titanosilicates, which are currently among the most efficient heterogeneous photocatalysts.^[18]^ They are characterized by a Si/Ti framework that combines concurrent tetrahedral and octahedral coordination. While the Al atom in the zeolite structure has a tetrahedral configuration, the Ti atom in the titanosilicates adopts an octahedral configuration [TiO_6_]^2−^ which is more stable.^[19]^ Titanosilicalites are also known examples of open structures, which exhibit high ionic conductivity. Usually, Si/Ti porous materials show good acid pH resistance and display very interesting photocatalytic properties. Well‐known examples of titanosilicates are the Engelhard Titanosilicate‐4 (ETS‐4) and Engelhard Titanosilicate‐10 (ETS‐10), developed by Engelhard Corporation.^[20]^ They are commonly referred for catalysis, ion exchange, and adsorption usages. ETS‐4 is a microporous material with a highly selective pore structure, ideal for gas separation, due to its thermal stability and ability to adjust pore size through “molecular sieving”.^[21]^ ETS‐10, on the other hand, is characterized by a larger pore structure compared to ETS‐4.^[20]^ It is a mesoporous material and contains linear chains of titanium oxide (TiO_6_) units within its silicate framework, giving rise to its unique properties. The larger pores and high ion‐exchange capacity of ETS‐10, are making it effective in applications like, water purification and heavy metal removal.^[22]^

The present research is focused on the synthesis of Zr modified ETS in which part of the Ti framework is replaced with Zr and their application for the photodegradation of the organic dyes Crystal violet and Methylene blue in model water solutions.

## Results and Discussion

The main goal of this work was to partially substitute *in situ* some of the Ti atoms with Zr in ETS‐4 in order to achieve enhanced photodegradation effect towards MB and CV. The selected synthesis protocol allowed us to produced monophasic Na−K‐ETS‐4 without the use of an organic structure‐directing agent.^[22]^ The recipe for obtaining Na−K‐ETS‐4 was already optimized. The question was would the synthesis protocol allow the replacement of Ti with Zr during the synthesis. Thus, in the synthesis protocol equimolar amounts of the TiCl_4_ were replaced with ZrCl_4_, all other parameters were kept unchanged: titanium source was TiCl_4_ dissolved in water, the silica source was amorphous fumed silica (nano silica) dissolved in a NaOH/KOH/water solution. In order to minimize the quartz formation, the temperature for synthesis of Na−K‐ETS‐4/xZr was kept at 200 °C (Scheme S1). The above considerations resulted in the synthesis of XRD pure Na−K‐ETS‐4/xZr forms (x=2.3, 6.3, 8.9 and 9.2 wt %). All samples obtained were characterized by WDXRF, PXRD, TG/DTA, FTIR and N_2_ adsorption/desorption for micro‐/mesoporosity, specific surface and pore size distribution (PSD). The capability of Na−K‐ETS‐4/xZr forms for photodegradation of CV and MB using white light was studied. The data concerning Na−K‐ETS‐10‐xZr are available in our previous studies.^[18c]^

### Physicochemical Characterization of Na‐K‐ETS‐4/xZr Form

The chemical composition of the obtained samples was investigated using WDXRF. According to the WDXRF results, although the initial synthesis included Zr amounts of 5, 15, 25, and 30 %, only approximately 2.3, 6.3, 8.9, and 9.2 wt % Zr were successfully incorporated into the framework of the final product (Table 1, 2). Additionally, the results indicate that ETS‐4 can sustain a maximum of 9.2 wt % Zr (starting amount 30 wt % Zr) without causing structural collapse. For simplicity, the sample labeling will reflect the incorporated wt % of Zr as determined by WDXRF, rather than the amounts used during synthesis.

**Table 1:** WDXRF‐Elemental analysis of the synthesized Na−K‐ETS‐4/xZr samples.

Samples	Na, wt %	K, wt %	Zr, wt %	Ti, wt %	Si, wt %
Na−K‐ETS‐4	20.37	7.20	–	23.14	49.30
Na−K‐ETS‐4/5Zr	16.76	8.89	2.3	30.61	41.75
Na−K‐ETS‐4/15Zr	15.20	8.92	6.3	27.80	41.80
Na−K‐ETS‐4/25Zr	15.30	10.10	8.9	24.80	41.00
Na−K‐ETS‐4/30Zr	14.76	10.41	9.2	23.25	43.36

**Table 2:** Chemical composition of the synthesized samples. The water content was calculated based on the TGA data.

Na−K‐ETS‐4‐x	Chemical composition
x=0.0 Zr	Na _8.03_ K _2.50_ (Ti _9.60_ Si _12.00_ O _38.00_ OH) x H_2_O _13.05_
x=2.3 Zr	Na _5.89_ K _1.84_ (Ti _5.16_ Zr _0.20_ Si _12.00_ O _38.00_ OH) x H_2_O_14.05_
x=6.3 Zr	Na _5.52_ K _2.11_ (Ti _4.36_ Zr _0.58_ Si _12.00_ O _38.00_ OH) x H_2_O_12.95_
x=8.9 Zr	Na _5.47_ K _2.12_ (Ti _4.26_ Zr _0.80_ Si _12.00_ O _38.00_ OH) x H_2_O_15.31_
x=9.2 Zr	Na _4.99_ K _2.07_ (Ti _3.78_ Zr _0.79_ Si _12.00_ O _38.00_ OH) x H_2_O_11.70_

**Figure 1:**
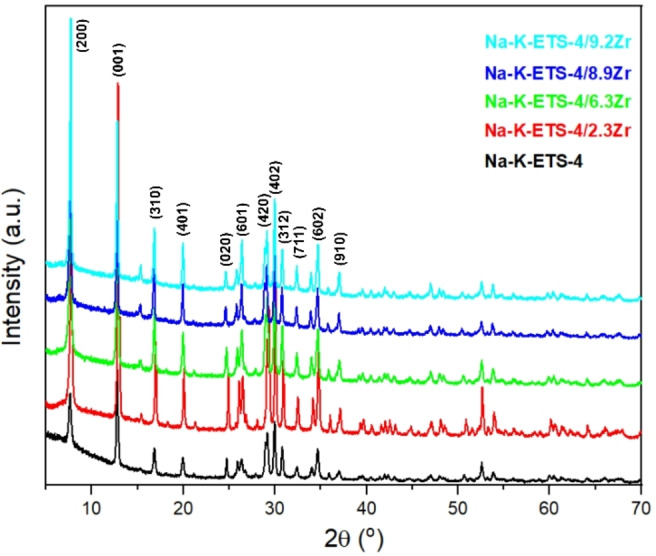
X‐ray powder diffraction of patterns of the studied samples.

The PXRD patterns of the Na−K‐ETS‐4 and Na−K‐ETS‐4/xZr (x=2.3, 6.3, 8.9 and 9.2 wt %) samples are shown in Figure 1. Starting Na−K‐ETS‐4 matched closely the ICDD pattern 01–074‐2754.^[23]^ At first glance, the PXRD patterns of the initial Na−K‐ETS‐4 and the partly Zr substituted Na−K‐ETS‐4 samples appear very similar, thus disclosing the framework conservation. However, a close inspection of the PXRD patterns reveals some relative intensity differences in the first two peaks ((200) and (001)) and also the appearance of diffraction peak at ~15° 2θ (400) for the ETS‐4/xZr samples that can be attributed to contractions/expansions of the small 6MR channels of ETS‐4. This conclusion is supported by the WDXRD data detecting that the increase of Zr content diminishes the amounts of Na in the ETS‐4 structure whereas the K amounts increases (Table 1). Moreover, an amorphous halo is present in the 25–35° 2θ region for the samples having 8.9 and 9.2 wt % Zr, suggesting the initiation of some structural changes with the increase of Zr content.

**Figure 2:**
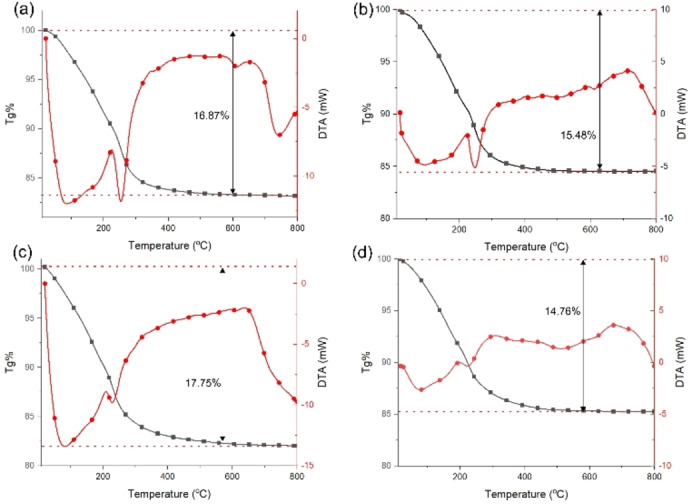
DTA‐TGA of Na−K‐ETS‐4/xZr, (a) x=2.3, (b) x=6.5, (c) x=8.9, and (d) x=9.2.

**Figure 3:**
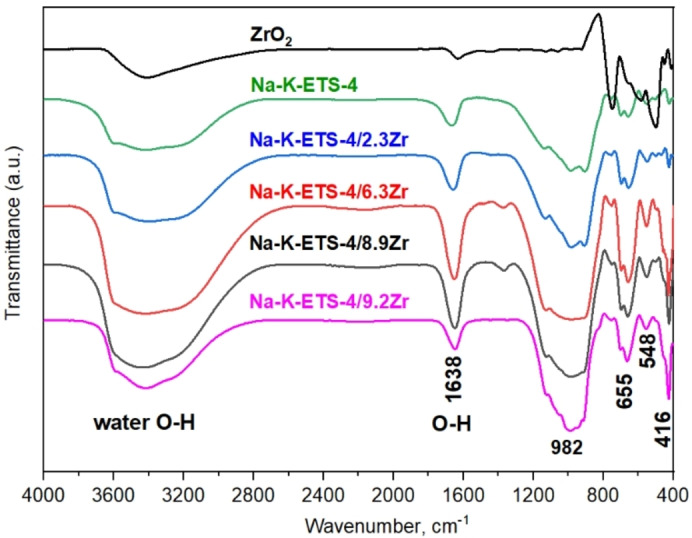
FTIR spectra of Na−K‐ETS‐4 and Na−K‐ETS‐4/xZr (x=2.3, 6.3, 8.9 and 9.2).

The thermal behavior of Na−K‐ETS‐4/ xZr (x=2.3, 6.3, 8.9 and 9.2 wt %) samples were studied by combined DTA‐TGA analyses in the temperature range 20–800 °C, using static air atmosphere (Figure 2). The total weight losses registered by the TGA vary between 14.76 wt % (for Na−K‐ETS‐4/9.2Zr) and 17.75 wt % (for Na−K‐ETS‐4/8.9Zr). The major weight losses are detected in the temperature range from 20–300 °C that can be assigned to the release of physisorbed or structural water.^[24]^ The release of the water molecules is accompanied by two overlapping endothermic effects (one broad and one sharp) with maxima at around 90 °C and 250 °C, respectively.^[25]^ After 300 °C only insignificant mass losses are registered. The DTA detects only one endothermic effect above 600 °C that could be related to complete amorphization or formation of new high temperature phases. The FTIR spectra of ZrO_2_, Na−K‐ETS‐4 and Na−K‐ETS‐4/xZr are displayed on Figure 3. The ZrO_2_ spectrum discloses four well discernable bands. A broad band encompassing the 3600–3000 cm^−1^ region and three sharper ones at ~1620, ~750 and ~490 cm^−1^. According to Khomenkova et al.^[26]^ the presence of the 750 cm^−1^ band evidences for the monoclinic ZrO_2_ (Baddeleyite).

With the exception of the broad band (3600–3000 cm^−1^ region) those bands are not observed in the Na−K‐ETS‐4/xZr spectra. Actually, the broad intensive bands observed in all spectra in the 3600–3100 cm^−1^ region are associated with the asymmetric O−H stretching vibrations of water molecules. The band centered at 1650–1630 cm^−1^ refers to characteristic vibration of H−O−H. The most intensive bands registered from Na−K‐ETS‐4/xZr samples are observed in the 1200–415 cm^−1^ region of the FTIR spectra. They are related to the framework vibrations of ETS‐4. More specifically, the bands in the 1100–910 cm^−1^ regions can be attributed to the stretching vibrations of Si−O−Si and Si−O−Ti. The band observed at 655 cm^−1^ is related to the Ti−O−Ti stretching. The intensity of the vibration at ~416 cm^−1^ steadily increases with Zr concentration, and is probably due to the Zr−O−Ti vibrations.

**Figure 4:**
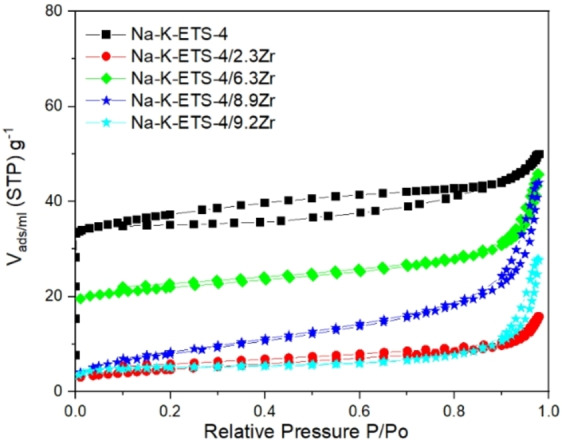
Nitrogen adsorption isotherms for Na−K‐ETS‐4 and and Na−K‐ETS‐4/xZr (x=2.3, 6.3, 8.9 and 9.2).

The nitrogen adsorption/desorption isotherms for Na−K‐ETS‐4 and Na−K‐ETS‐4/xZr samples are shown in Figure 4, and the textural parameters determined from analyzing the isotherms are listed in Table 3. In Figure 4, one can clearly see that the N_2_ adsorption/desorption isotherms for Na−K‐ETS‐4 possess a microporous part, while the samples Zr modified samples, Na−K‐ETS‐4/xZr, lack microporous adsorption. The N_2_ adsorption/desorption isotherms of Na−K‐ETS‐4 and Na−K‐ETS‐4/xZr can be classified as of type I and type II, respectively, according to the IUPAC classification.^[27]^ Moreover, the N_2_ adsorption/desorption isotherms of Na−K‐ETS‐4 and Na−K‐ETS‐4/xZr demonstrate H3 and H4 type hysteresis loop indicating the respective micro‐/mesoporous and mesoporous nature of the materials. The specific surface area (SSA) of the samples is in the interval between 16.45 and 166.37 m^2^/g. The mesoporous diameters due to textural microporosity were found in the range of 3.7–7.9 nm.

**Table 3:** Textural parameters for Na−K‐ETS‐4 and Na−K‐ETS‐4/xZr as deduced from Nitrogen adsorption isotherms.

Samples	SBET m^2^.g^−1^	Vt cm^3^.g^−1^	Pore diameter nm
Na−K‐ETS‐4	166.37	0.075	5.4
Na−K‐ETS‐4/2.3Zr	16.45	0.022	5.3
Na−K‐ETS‐4/6.3Zr	68.65	0.063	3.7
Na−K‐ETS‐4/8.9Zr	29.40	0.058	7.9
Na−K‐ETS‐4/9.2Zr	17.11	0.037	5.5

**Figure 5:**
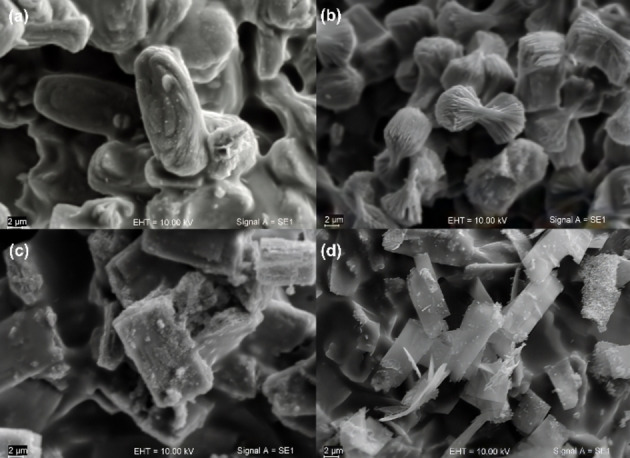
SEM images of (a) Na−K‐ETS‐4, (b) Na−K‐ETS‐4/2.3Zr (c) Na−K‐ETS‐4/6.3Zr and (d) Na−K‐ETS‐4/9.2Zr.

SEM images show that Na−K‐ETS‐4 and Na−K‐ETS‐4/2.3Zr crystallizes as lamellar aggregates (Figure 5a and b). With the increase of Zr content a more pronounced block‐shaped aggregates appear (Figure 5c). The morphology of the Na−K‐ETS‐4/9.2Zr is composed of prismatic block‐shaped crystals and thinner plates (Figure 5d). The size of the crystalline aggregates is in all cases around 4–6 μm in length and 2–3 μm width.

### Photodegradation of CV and MB

**Figure 6:**
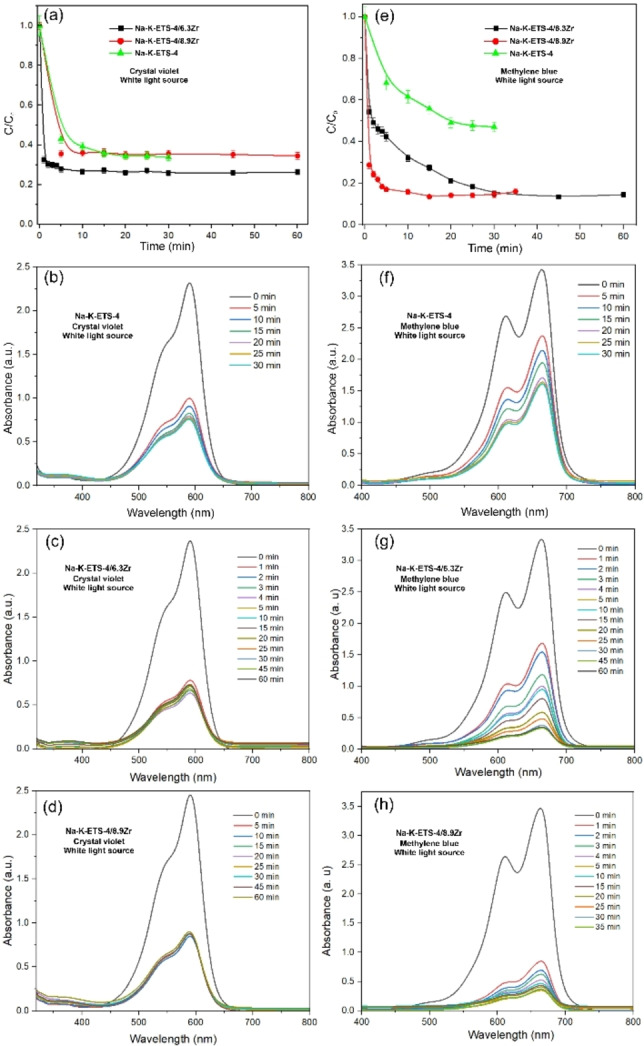
Photodegradation graph of CV and MB for Na−K‐ETS‐4/6.3Zr and Na−K‐ETS‐4/8.9Zr samples.

The synthesized materials were tested for their efficiency in the photodegradation of the dyes crystal violet (CV) and methylene blue (MB). The photocatalytic conversion of CV and MB using different Na−K‐ETS‐4/xZr samples as catalysts was performed in aqueous media and under visible light irradiation (λ>420 nm). The absorbance of the CV and MB dye solutions gradually decreased in the presence of a photocatalyst and visible light irradiation. Only those Na−K‐ETS‐4/xZr (x=6.3 and 8.9) samples that demonstrated the best effectiveness are shown in Figure 6. Figure 6a displays that Na−K‐ETS‐4/6.3Zr achieves the best photodegradation efficiency for CV (~76.6 % Figure 6a, black) in comparison with starting Na−K‐ETS‐4 and the Na−K‐ETS‐4/8.9Zr samples (~61 % efficiency, Figure 6a green and red). For MB (Figure 6e), the photodegradation efficiencies of Na−K‐ETS‐4/6.3Zr (Figure 6e, black) and Na−K‐ETS‐4/8.9Zr (Figure 6e, red) are comparable (~87 %). However, the time required to reach this efficiency is clearly in favor of Na−K‐ETS‐4/8.9Zr (10 minutes vs. 30 minutes for Na−K‐ETS‐4/6.3Zr). These findings indicate that the photocatalytic activity for CV and MB using Na−K‐ETS‐4/xZr as a catalyst are time‐dependent.^[28]^ Nevertheless, both Na−K‐ETS‐4/6.3Zr and Na−K‐ETS‐4/8.9Zr demonstrate higher efficiency than the unmodified Na−K‐ETS‐4 (~50 % efficiency, Figure 6e, green).

**Figure 7:**
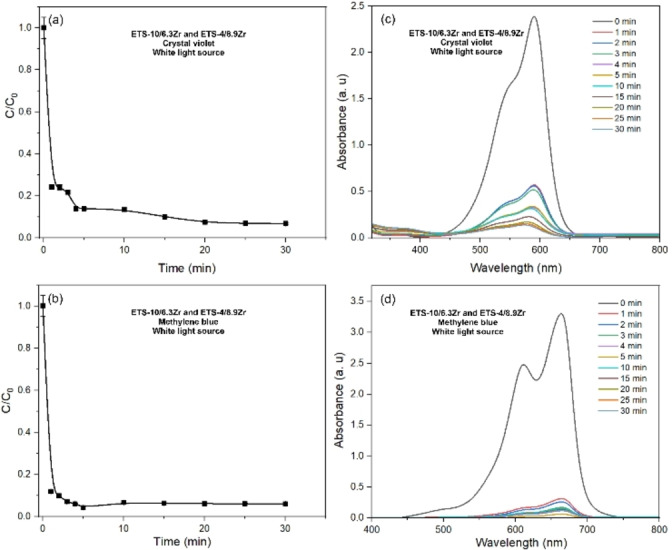
Photodegradation efficiency of CV and MB combining Na−K‐ETS‐10/6.3Zr and Na−K‐ETS‐4/8.9Zr.

The data regarding the photodegradation efficiency of Na−K‐ETS‐10 towards CV and MB is shown on Figure S2. The data reveals that Na−K‐ETS‐10 has 67 % and 85 % efficiency towards CV and MB respectively. However, the photodegradation kinetics are quite slower when compared to the Na−K‐ETS‐4/xZr, reaching a plateau after ~50 minutes. Thus in order to further improve the catalysts efficiency and kinetics, we attempted a combination of catalysts featuring Zr modified Na−K‐ETS‐4 and Na−K‐ETS‐10^[18c]^ in 1 : 1 weight ratio (Figure 7). The results indicate that the photocatalytic degradation efficiency for CV and MB using a combination of Na−K‐ETS‐10/6.3Zr and Na−K‐ETS‐4/8.9Zr is 90.3 % and 96.5 %, respectively. The combination of Na−K‐ETS‐10/6.3Zr and Na−K‐ETS‐4/8.9Zr enhances both the efficiency and the effectiveness of the photodegradation process, particularly for MB solutions. For MB, it is evident that this combination of catalysts achieves a photodegradation efficiency plateau of approximately 96.5 % after just 5 minutes of irradiation. For CV, although the efficiency also improves to around 90 %, the kinetics of the process remain unchanged. The diminishing intensity of the peaks at 590 and 663 nm for CV and MB dyes respectively along with the discoloration of the solution suggests the decomposition of CV and MB proceeds towards CO_2_ and H_2_O.^[29]^

### Catalyst Recycling

**Figure 8:**
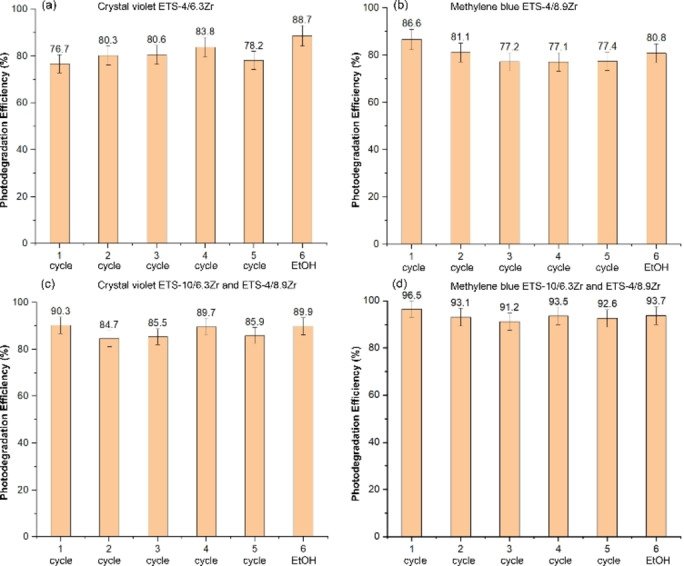
Reusability and regeneration potential of Na−K‐ETS‐4/xZr for (a, c) crystal violet and (b) methylene blue; the sixth cycle discloses the catalyst recovery achieved with simple ethanol wash.

Catalysts recycling is an important economical factor for practical applications mainly related to their stability and performance. In this work, recycling studies were conducted using Na−K‐ETS‐4/xZr (x=6.3 % and 8.9 %) and the combination ETS‐10/6.3Zr with Na−K‐ETS‐4/8.9Zr as selective photocatalysts for the degradation of crystal violet and methylene blue over five successive cycles. At the end of each cycle, the catalysts were centrifuged, thoroughly washed with deionized water, dried and reused in the next cycle (Figure 8). In general, only a slight decrease in photocatalytic degradation efficiency for CV and MB was observed (~±10 %) with repeated use of all studied catalysts. For example, the photodegradation efficiency of Na−K‐ETS‐4/6.3Zr versus CV remains constant (~78 %, Figure 8a) while for Na−K‐ETS‐4/8.9Zr versus MB the decrease of the photodegradation efficiency is minimal, from 86.6 % in the first cycle to 77.4 % in the fifth cycle (Figure 8b).

In the case of the combination of two types of Zr modified Na−K‐ETS‐10/6.3Zr and Na−K‐ETS‐4/8.9Zr, the decrease of efficiency for both CV and MB were only 4 % from the first to the fifth cycle (Figure 8c and d). Deviation from this trend is observed only for the Na−K‐ETS‐4/6.3Zr catalyst against MB, where the phododegradation efficiency decreased from 86.6 % in the first cycle to 54.6 % in the fifth cycle (Figure S1). After five consecutive cycles, the catalysts were centrifuged, washed with ethanol, and dried at 60 °C, restoring their initial efficiency (Figure 8a–d, sixth cycle). Interestingly, the washing of Na−K‐ETS‐4/6.3Zr with ethanol increased the efficiency with 12 % towards CV, compared to the initial sample.

**Figure 9:**
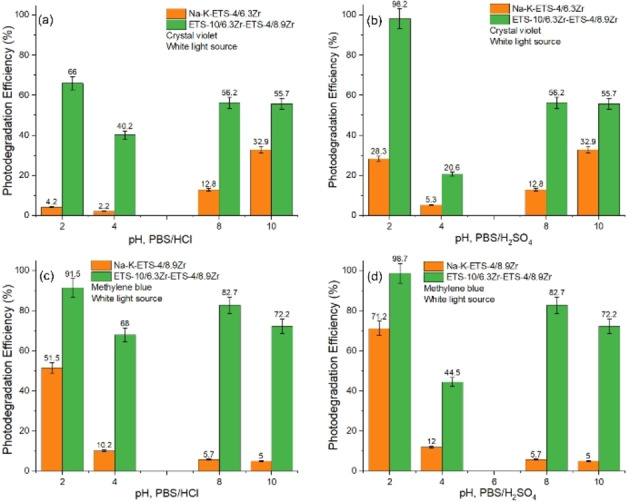
The effect of pH photodegradation efficiency (%) process of (a, b) CV and (c, d) MB.

Alongside the recycle and reuse of catalysts, their efficacy towards the studied process at different pH levels is a significant factor for practical applications. Thus, the effect of pH on the photodegradation of CV was studied for Na−K‐ETS‐4/6.3Zr and the combination Na−K‐ETS‐10/6.3Zr and Na−K‐ETS‐4/8.9Zr. For MB the selection was Na−K‐ETS‐4/8.9Zr its combination with Na−K‐ETS‐10/6.3Zr. The chosen pH levels for the efficiency studies involved acidic (pH 2 and 4) and alkaline (pH 8 and 10) ones. The adjustment of the pH was carried out using either H_2_SO_4_ or HCl. From the results one can see that the pH changes are more pronounced when only one type of catalyst is employed e. g. Na−K‐ETS‐4/6.3Zr or Na−K‐ETS‐4/8.9Zr. The efficiency drops significantly (generally below 30 %) the only exception is for MB at pH 2 and H_2_SO_4_ (Figure 9d). When the combination of the catalysts is employed (Na−K‐ETS‐10/6.3Zr and Na−K‐ETS‐4/xZr) the loss of efficiency is not as pronounced. The results suggest that the loss of efficiency is almost double for the alkaline pH. Interestingly at lower pH e. g. pH 2, the efficiency is improved.

**Figure 10:**
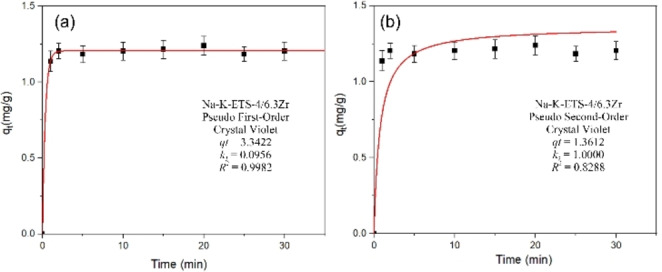
Kinetic model fit for CV photodegradation by Na−K‐ETS‐4/6.3Zr using (a) Non‐linear PFO and (b) Non‐linear PSO; the inset represent the parameters obtained from the fitting.

**Figure 11:**
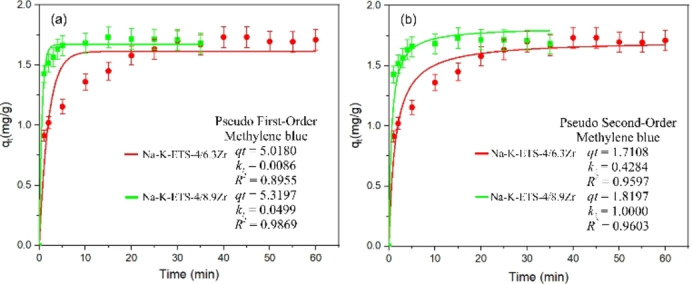
Kinetic model fit for MB photodegradation by Na−K‐ETS‐4/6.3Zr (red) and Na−K‐ETS‐4/8.9Zr (green) using (a) Non‐linear PFO and (b) Non‐linear PSO; the inset represent the parameters obtained from the fitting.

**Figure 12:**
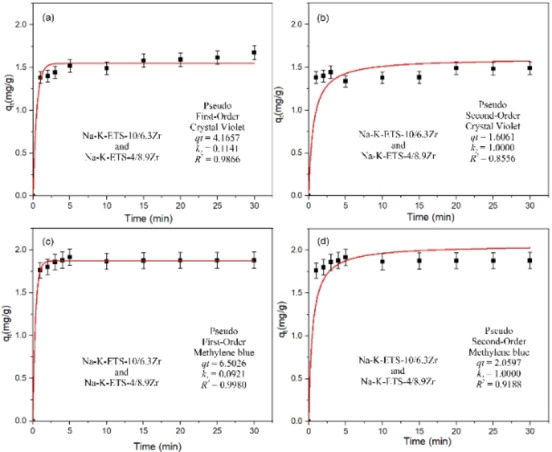
Kinetic model fit for CV and MB photodegradation by combination of Na−K‐ETS‐10/6.3Zr and Na−K‐ETS‐4/8.9Zr using (a) Non‐linear PFO for CV, (b) Non‐linear PSO for CV, (c) Non‐linear PFO for MB and (d) Non‐linear PSO for MB; the inset represent the parameters obtained from the fitting.

The kinetics of the photodegradation process were studied using nonlinear pseudo‐first‐order and pseudo‐second‐order models (Figures 10–11, 12). The selection of those models was made following Tan and Hameed^[30]^ suggestion that nonlinear modeling is the better technique compared to linear regression as it provides more realistic kinetic parameters. Compared to the linear PFO and PSO the advantage of nonlinear modelling is the use of a single fitting parameter: the predefined objective (OF),[^30^, ^31^] thus facilitating the estimation of model parameters. The drawback of the nonlinear modelling is the requirement and prior knowledge of *q_t_*, which makes these models unsuitable for modeling photodegradation systems below equilibrium. The results for nonlinear PFO and PSO are shown on Figure 10 for CV or MB photodegradation using Na−K‐ETS‐4/6.3Zr. The results of nonlinear PFO and PSO fitting for CV or MB photodegradation using Na−K‐ETS‐4/6.3Zr or Na−K‐ETS‐4/8.9Zr are shown Figure 11, and the nonlinear PFO and PSO fits for the combination ETS‐10/6.3Zr and ETS‐4/8.9Zr are shown in on Figure 12.

From the obtained results it is clear that all synthesized materials conform to Non‐linear Pseudo‐First‐Order kinetic model with *R* ^*2*^ 0.998, 0.9869, 0.9866 and 0.9980 (Figures 10–12). This means that the rate of degradation is directly proportional to the concentration of the degrading species CV or MB e. g. the degradation rate depends linearly on the concentration of the reactant. The mechanism of photodegradation likely involves a single rate‐determining step probably the concentration of the reactants (CV or MB) in solution. The interaction between Na−K‐

**Figure 13:**
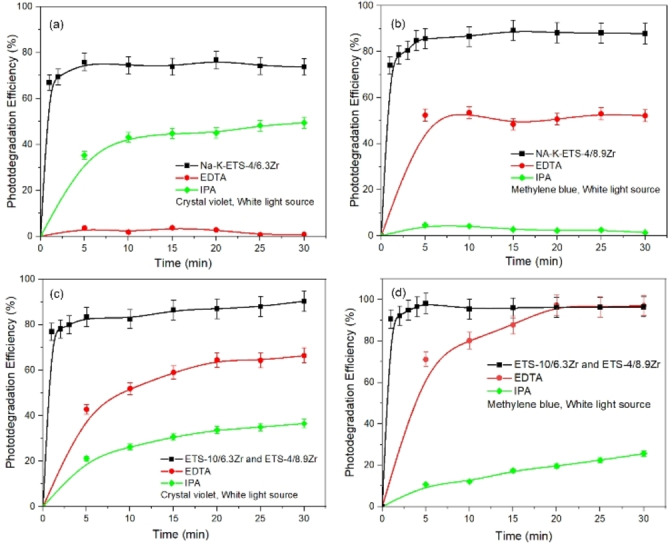
Effect of EDTA and IPA scavengers on the degradation process of (a, c) CV and (b, d) MB.

ETS‐4/6.3Zr and CV or MB molecules likely occurs on the surface of the photocatalyst when these molecules are present in the solution. Thus, the surface properties of the catalysts can be related to the adsorption capacity and the generation of reactive species. Usually, a higher surface area and presence of defects produce enhanced photocatalytic efficiency. According to the data reported in Table 3, Na−K‐ETS‐4/6.3Zr surface area is the highest (~68 m^2^ g^−1^), followed by Na−K‐ETS‐4/8.9Zr (~29 m^2^ g^−1^). Those data correlate with the observed photodegradation efficiency. To explore the active species in the photocatalytic reaction process involving Na−K‐ETS‐4/xZr or its combination with Na−K‐ETS‐10/xZr for CV and MB, isopropanol (IPA) and ethylenediaminetetraacetic acid (EDTA) were used as scavengers for superoxide radicals (⋅O_2_^−^), hydroxyl radicals (⋅OH) and photogenerated holes (h^+^).^[32]^ The results are shown in Figure 13. According to the results of the scavenging experiments, the degradation of crystal violet and methylene blue by the catalysts is significantly reduced after the addition of the above‐mentioned scavenging agents in the photocatalytic process, indicating that ⋅O_2_^−^ and ⋅OH are the main reactive groups in the photocatalytic degradation of the dyes by Na−K‐ETS‐4/xZr. The electrons can better interact with the O_2_ in the solution to generate the ⋅O_2_^−^ ion. While the photogenerated holes interact with the H_2_O to generate ⋅OH.[^29^, ^33^] For comparison the photodegradation efficiencies of other catalysts toward MB and CV taken from the literature are provided in Table 4. The values of the reported efficiencies of Na−K‐ETS‐4/xZr compete with the most promising candidates.

**Table 4:** Comparative degradation efficiencies of various catalysts toward CV and MB.

Catalyst	Degradation Efficiency (%)		
	CV	MB	Ref.
Hematite‐HA composite	97.0		^[4]^
BiVO_4_/FeVO_4_	100.0		^[28a]^
1 T‐MoS_2_@(Bi_0.40_Fe_0.60_)VO_4_	99.9		^[28b]^
ACL/Fe_3_O_4_	98.3		^[5b]^
TNTs	75.0		^[32b]^
Cloisite 30B/ZnO/Ag_2_O	99.2	98.4	^[6f]^
Dandelion‐ZnS		88.0	^[7]^
TiO_2_		76.0	^[9]^
ZnO_100_		29.0	^[9]^
Ag_2_S@TiO_2_ NFs		97.0	^[12]^
β‐Cu_2_V_2_O_7_/Zn_2_V_2_O_6_ (CZVO)		74.9	^[13a]^
BiVO_4_		91.9	^[13c]^
InVO_4_–BiVO_4_		70.0	^[13e]^
Nanosheets g‐C_3_N_4_/CNM^BGt^		77.0	^[14c]^
CN−G‐0.4 was decomposed on pristine g‐C_3_N_4_		100.0	^[14d]^
magnetite nanoparticles		93.4	^[15a]^
Fe_3_O_4_/Ag_6_Si_2_O_7_		98.0	^[15b]^
Serpentine		90.5	^[34]^
Alluvium		98.2	^[5a]^
ZnO@C07		99.8	^[32a]^
Na−K‐ETS‐4/6.3Zr	76.7	86.6	This work
Na−K‐ETS‐4/8.9Zr	60.8	86.6	This work
Na−K‐ETS‐10/6.3Zr‐ and Na−K‐ETS‐4/8.9Zr	90.3	96.5	This work

Diffused reflectance spectroscopy (DRS) was used to determine the bandgap of as‐synthesized samples. The bandgap is calculated from the diffused reflectance spectrum using the Kubelka–Munk theory.^[35]^ (This is the missing ref. [35])The graph is plotted between *hν* on the x‐axis and [F(R)hν]^2^ on the *y*‐axis by extrapolating the linear fitted region on the *x*‐axis. The obtained band gaps are shown in Table 5. The *Eg* values correspond to a wide band gap catalyst (Figures S3–S10). However, the values of Na−K‐ETS‐4/6.3Zr and Na−K‐ETS‐10/6.3Zr/Na−K‐ETS‐4/8.9Zr are comparable thus the improved efficiency is probably due to the difference in the structures of the two ETS and thus producing different surface/pore characteristics and active centers.

**Table 5:** Energy band gap for Na−K‐ETS‐10, Na−K‐ETS‐4, Na−K‐ETS‐4/xZr, ZrO_2_ and Na−K‐ETS‐10/6.3Z/Na−K‐ETS‐4/8.9Zr samples obtained from Tauc plots, Figures S3–S10.

Samples	Energy (eV)
Na−K‐ETS‐10	4.41
Na−K‐ETS‐4	4.24
Na−K‐ETS‐4/2.3Zr	4.27
Na−K‐ETS‐4/6.3Zr	4.32
Na−K‐ETS‐4/8.9Zr	4.34
Na−K‐ETS‐4/9.2Zr	4.32
Na−K‐ETS‐10/6.3Zr/Na−K‐ETS‐4/8.9Zr	4.31
ZrO_2_	5.22

## Conclusions

Na−K‐ETS‐4/xZr materials with partial substitution of Ti by Zr in the framework were successfully synthesized using the hydrothermal technique, without the use of structure‐directing agents or templates. The Na−K‐ETS‐4/xZr structure was isotypical to the Na−K‐ETS‐4 one and was conserved with the increased the amounts of Zr. The limit of Zr/Ti substitution was around 9.2 wt %. The N_2_ adsorption/desorption isotherms exhibited mesoporous features for Na−K‐ETS‐4/xZr vs microporous for Na−K‐ETS‐4. Based on the photodegradation results obtained in this work, it was concluded that Na−K‐ETS‐4/xZr zeolites are efficient materials for the degradation of the methylene blue and crystal violet dyes present in aqueous solutions. Photodegradation of CV and MB reached 90.3 and 96.5 % efficiency respectively when the combination Na−K‐ETS‐10/6.3Zr‐ and Na−K‐ETS‐4/8.9Zr was employed. The ETS catalysts showed good reusability after five cycles. The photodegradation efficiency of MB displayed a slight drop from 86.6 % to 77.4 % when for Na−K‐ETS‐4/8.9Zr. For the combination of Na−K‐ETS‐10/6.3Zr with Na−K ETS‐4/8.9Zr for CV and MB we have a small drop of efficiency, 90.3–85.9 and 96.5–92.6, respectively. After the fifth cycle a complete regeneration of efficiency was achieved after a simple ethanol wash of the catalysts. The kinetic studies showed that the removal of CV and MB was a rapid process, which obeyed the non‐linear pseudo‐first‐order model. The results suggest that Na−K‐ETS‐4/xZr possess an excellent potential for degrading organic pollutants present in water.

## Conflict of Interests

There are no conflicts to declare.

1

## Supporting information

As a service to our authors and readers, this journal provides supporting information supplied by the authors. Such materials are peer reviewed and may be re‐organized for online delivery, but are not copy‐edited or typeset. Technical support issues arising from supporting information (other than missing files) should be addressed to the authors.

Supporting Information

## Data Availability

The data that support the findings of this study are available from the corresponding author upon reasonable request.
