# Supplementary material for: Elaboration and Characterization of Different Zirconium Modified ETS Photocatalysts for the Degradation of Crystal Violet and Methylene Blue
Source: ChemistryOpen. 2024 Nov 13;14(3):e202400348. doi: 10.1002/open.202400348 (PMC12128148; doi:10.1002/open.202400348)
Supplement: Supplementary file 1 — Supporting Information [file OPEN-14-e202400348-s001.pdf]

ChemistryOpen

Supporting Information

Elaboration and Characterization of Different Zirconium Modified ETS Photocatalysts for the Degradation of Crystal Violet and Methylene Blue

Hristina I. Lazarova,* Rusi I. Rusew, Liliya V. Tsvetanova, Borislav Z. Barbov, Elena S. Tacheva, and Boris L. Shivachev*

Supporting Information

Elaboration and characterization of Different Zirconium modified ETS Photocatalysts for the Degradation of Crystal Violet and Methylene Blue

Hristina I. Lazarova, *[a] Rusi I. Rusew [a], Liliya V. Tsvetanova [a], Borislav Z. Barbov [a], Elena S. Tacheva [a] and Boris L. Shivachev [a]

[a] Institute of mineralogy and crystallography "Acad. Ivan Kostov" – Bulgarian academy of sciences (IMC-BAS), Acad. G. Bonchev Str., Bl. 107, 1113 Sofia, Bulgaria here.

Corresponding author: Hristina I. Lazarova, lazarova@imc.bas.bg

Contents

Experimental 2

Scheme S1. Depiction of the Na-K-ETS-4/xZr synthesis steps; the * denotes the addition of appropriate amounts of ZrCl4. 2

Scheme S2. General photocatalytic degradation setup and experimental timeline. 5

Figure S1. Reusability and regeneration potential of Na-K-ETS-4/6.3Zr for MB. 5

Figure S2. Photodegradation kinetics for Crystal violet (a) and Methylene blue (b) using Na-K-ETS-10 as catalyst under white light radiation. 6

Figure S3. Tauc plot for Na-K-ETS-4/2.3Zr. 7

Figure S4. Tauc plot for Na-K-ETS-4/6.3Zr. 8

Figure S5. Tauc plot for Na-K-ETS-4/8.9Zr. 9

Figure S6. Tauc plot for Na-K-ETS-4/9.2Zr. 10

Figure S7. Tauc plot for Na-K-ETS-10/6.3Zr / Na-K-ETS-4/8.9Zr. 11

Figure S8. Tauc plot for ZrO_2. 12

Figure S9. Tauc plot for Na-K-ETS-4 13

Figure S10. Tauc plot for Na-K-ETS-10 14

Experimental

Materials

All starting reagents for the synthesis of Na-K-ETS-4, Na-K-ETS-4/xZr forms and those used for photocatalytic activity experiments were purchased from Sigma Aldrich (Schnelldorf, Germany), Alfa Aesar (Heysham, UK) or Honeywell (Charlotte, North Carolina, US) and used without additional purification. The reagents used included nanosized silicon dioxide (Fumed SiO2, 0.2–0.3 µm aggregates, Sigma Aldrich), titanium tetrachloride (TiCl4, 99.6%, Alfa Aesar), sodium hydroxide (NaOH, 97%, Sigma Aldrich), potassium hydroxide (KOH, >85%, Honeywell]), Zirconium (IV) chloride (ZrCl4, 99.9%, Sigma Aldrich), methylene blue hydrate (>97%, Sigma Aldrich), crystal violet (97%, Sigma Aldrich). The water used was ultrapure water (electrical conductivity—0.055 µS/cm, Q-FRONT EDI/BIO, Adrona, Riga, Latvia).

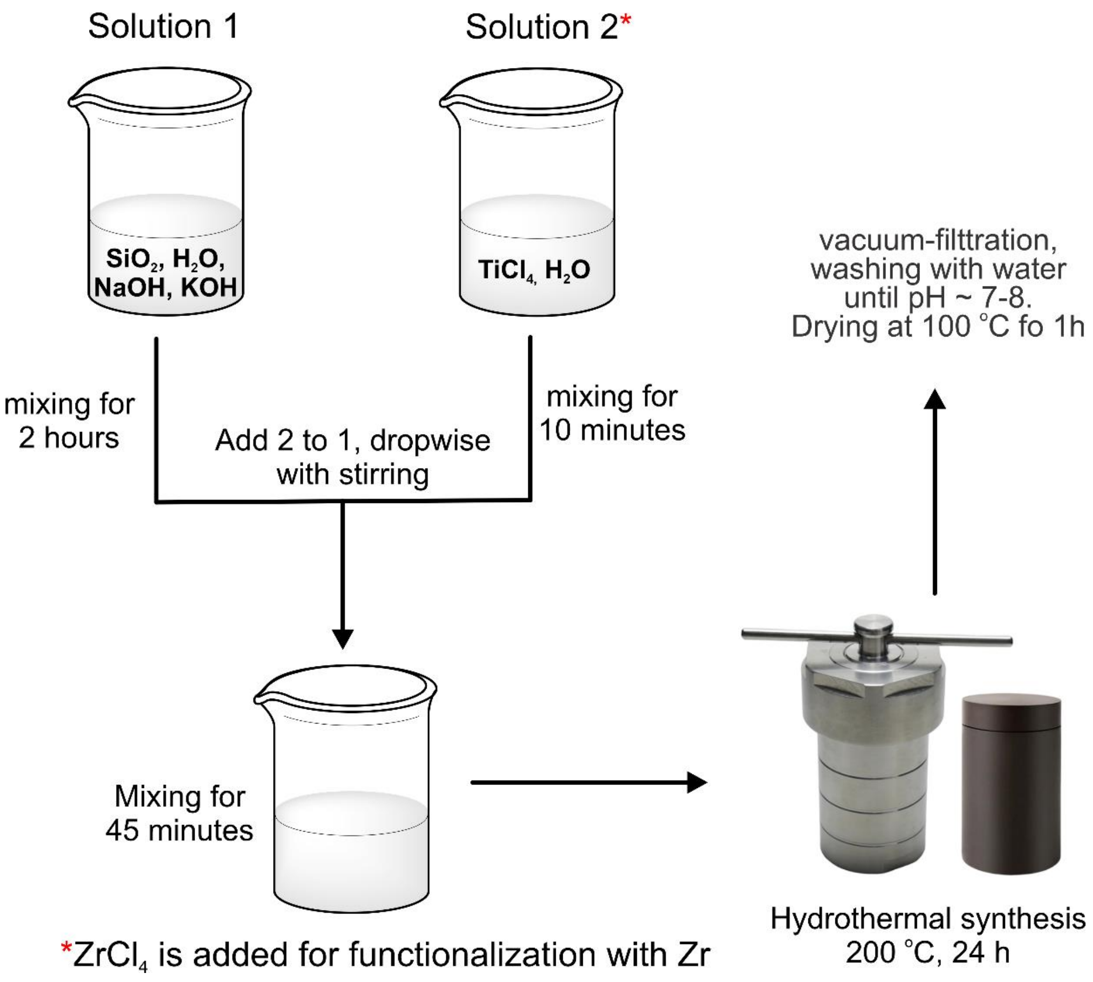

Scheme S1. Depiction of the Na-K-ETS-4/xZr synthesis steps; the * denotes the addition of appropriate amounts of ZrCl4.

Synthesis

The parent titanosilicate Na-K-ETS-4 was synthesized from a suspension with a molar composition 9.27 Na_2O:0.92 K_2O:2.33 TiO_2:10 SiO_2:675 H_2O. The reagents are added in the following sequence: Solution 1: 16.48 g of sodium hydroxide, 2.70 g of potassium hydroxide, and 13.35 g of silicon dioxide (fumed), were added to 180 mL of distilled water, and the mixture was homogenized for 2 h with stirring. Solution 2: 5.42 mL of titanium tetrachloride was added to 90 mL of distilled water, and the mixture was homogenized mechanically for 10 min. After that, solution 2 was added to solution 1 under constant stirring; the resulting white gel mass was re-homogenized with stirring for 45 min of room temperature. The crystallization was performed in stainless-steel Teflon-lined autoclave under autogenous static conditions for 24 h at 200ºC. The obtained product was vacuum-filtered and washed several times with distilled water until the pH reached ~7–8. After that, the product was dried at 100ºC for 1h. The Na-K-ETS-4/xZr was synthesized according to the procedure described in 2.1.1. The difference in the synthesis procedure was that the amounts (moles) of added $ZrCl_4$ were subtracted from the amounts of $TiCl_4$ (2.33 – x). The molar composition of gel was 9.27 Na_2O:0.92 K_2O:x$ZrCl_4$:(2.33 – x) TiO_2:10 SiO_2:675 H_2O, where x = 0.12, 0.35, 0.58 and 0.70. The synthesis steps are visualized in Scheme S1.

Wavelength Dispersive X-ray Fluorescence (WDXRF) Spectroscopy

The chemical composition of the synthesized samples was determined on a Supermini200 WDXRF spectrometer (Rigaku, Tokyo, Japan). Data collection was performed at 50 kV and 4.00 mA. Each sample was crushed and then pressed to obtain a tablet. The sample/tablet was placed

in a holder with an irradiated area of 30 mm in diameter. The weight ratio of the amount of sample to the amount of glue (Acrawax C powder) was 5:1 A semi-quantitative method (SQX) was used to determine the elemental composition.

Powder X-ray diffraction (PXRD) analysis

Powder X-ray diffraction (PXRD) analysis was used for monitoring the syntheses results and product's purity via phase identification. Powder X-ray diffraction analysis was performed on Empyrean (MalvernPanalytical, Almelo, The Netherlands) diffractometer equipped with a PIXcel3D detector and copper X-ray source (Cu$K\alpha$ = 1.5406 Å). The diffracting patterns were collected in the 3-70° 2θ range, under operating conditions of 40 kV and 30 mA and step size of 0.013°.

Specific surface area (SSA) analysis - N2 physisorption

The specific surface area and porosity of Na-K-ETS-4 and Zr modified Na-K-ETS4 was analyzed using 3Flex analyzer (Micromeritics, Norcross, GA, USA). Before analysis, the samples were degassed in situ at 80°C for 5 h under vacuum (>1.10^{-6} mmHg). The physisorption experiments were carried out under liquid nitrogen (77K) using N_2 probe molecule. Quantitative information for the specific surface area (S, $m^2.g^{-1}$), micropore volume (V m, $cm^3.g^{-1}$), pore size distribution, etc., were obtained by analyzing the resulting N_2 adsorption / desorption isotherms. Brunauer, Emmett and Teller (BET) specific surface areas were calculated from adsorption data in the

relative pressure range from 0.05 to 0.31 . The total pore volume was estimated based on the amount adsorbed at a relative pressure of 0.96 . Micropore volumes were determined using the *t*-plot method using Harkins and Jura equation and the Horvath-Kawazoe methods . Pore size distributions (PSDs) were calculated from nitrogen adsorption data using an algorithm based on the ideas of Barrett, Joyner and Halenda (BJH) . The mesopore diameters were determined as the maxima on the PSDs for the given samples.

Fourier Transform Infrared (FTIR) Spectroscopy

The FTIR spectra of the samples were recorded on a Tensor 37 (Bruker, Berlin, Germany) spectrometer using KBr pellets. For each sample, 128 scans were collected at a resolution of 2 cm^{-1} over the wavenumber region 4000-400 cm^{-1}.

Thermal Analysis

The thermal behavior of the Na-K-ETS-4 and Na-K-ETS-4/xZr samples obtained in this study was evaluated through differential thermal analysis (DTA) combined with thermogravimetric analysis (TGA). The phase transitions and weight evolution upon heating (5 K/min) were obtained in static air atmosphere on samples weighing around 20 mg in the 25–800°C temperature range using Al_2O_3 ceramic crucibles on a SETERAM Setsys Evolution (Sophia Antipolis, France) analyzer.

Scanning electron microscopy (SEM)

Scanning electron microscopy (SEM) of carbon coated Na-K-ETS-4/xZr samples was conducted on ZEISS SEM EVO 25 LS (Germany) in conjunction with energy dispersive X-ray spectroscopy (EDAX Trident) in secondary electron (SE) imaging regime. The accelerating voltage was 10 kV, I ~1 nA and the pressure was of the order of 10^{-5} Pa.

Photocatalytic Degradation Experiments

The effective photocatalytic degradation of the organic dyes - crystal violet and methylene blue - in the presence of Zr-substituted Na-K-ETS-4 was evaluated using UV-Vis spectroscopy. The details of the experiments were as follows: 50 mg of Na-K-ETS-4/xZr or Na-K-ETS-10/xZr was added to 10 mL of a 10 µg/mL CV or 20 µg/mL MB dye solution. A volume of 2 mL was taken from the reaction mixture at different time intervals (t = 1, 2, 3, 4, 5, 10, 15…. min etc.) and centrifuged at 14000 rpm for 1 min. Next, the kinetics of the photodegradation reaction were examined by measuring the absorption spectra using spectrophotometer Cary 4000 (Agilent, Santa Clara, CA, USA). The decrease in dye concentration was examined until ΔC = constant by measuring the characteristic absorbance peak at λ_{max} = 590 nm for CV and λ_{max} = 663 nm for MB. The efficiency of photodegradation was determined by extrapolation from calibration curves using the following equation (eqn (1):

$$\text{Degradation Efficiency (\%)} = \frac{(C_0 - C)}{C_0} \text{ x } 100 \qquad (1)$$

where C_0 and C are the solution concentration at t = 0 and after some irradiation time.

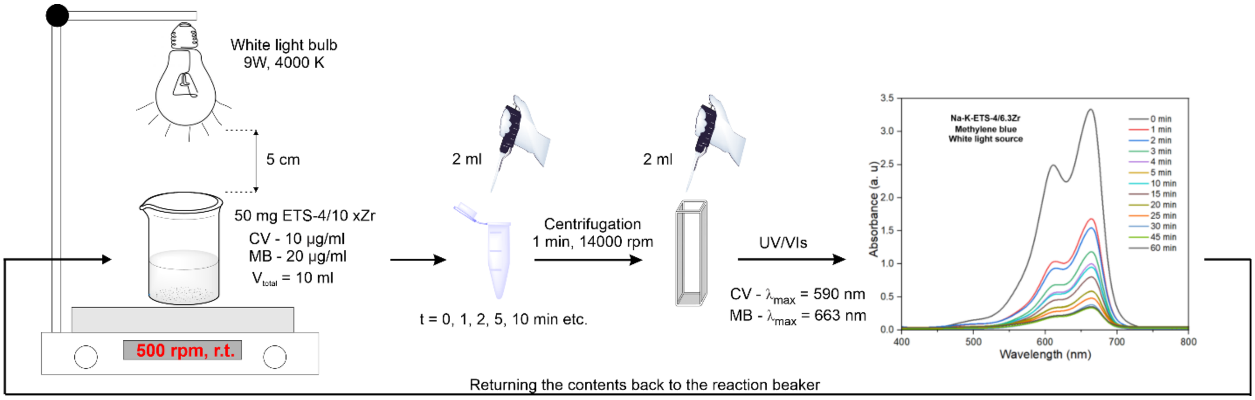

Scheme S2. General photocatalytic degradation setup and experimental timeline.

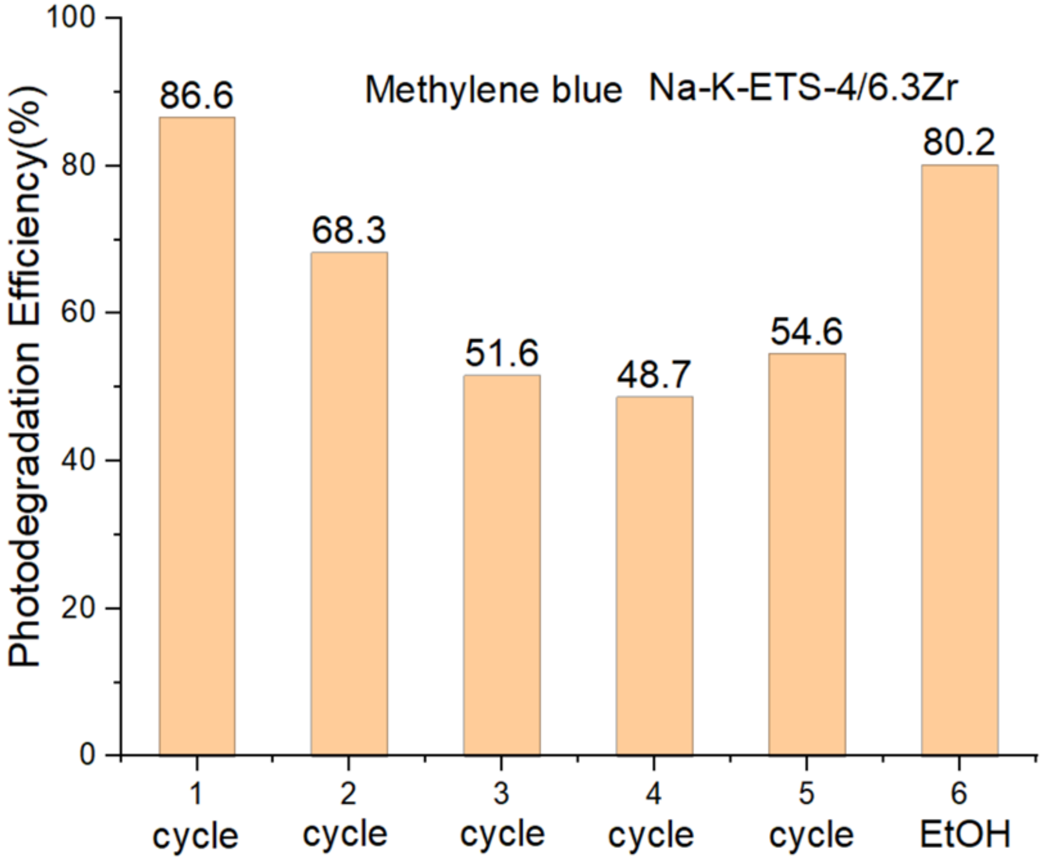

Figure S1. Reusability and regeneration potential of Na-K-ETS-4/6.3Zr for MB.

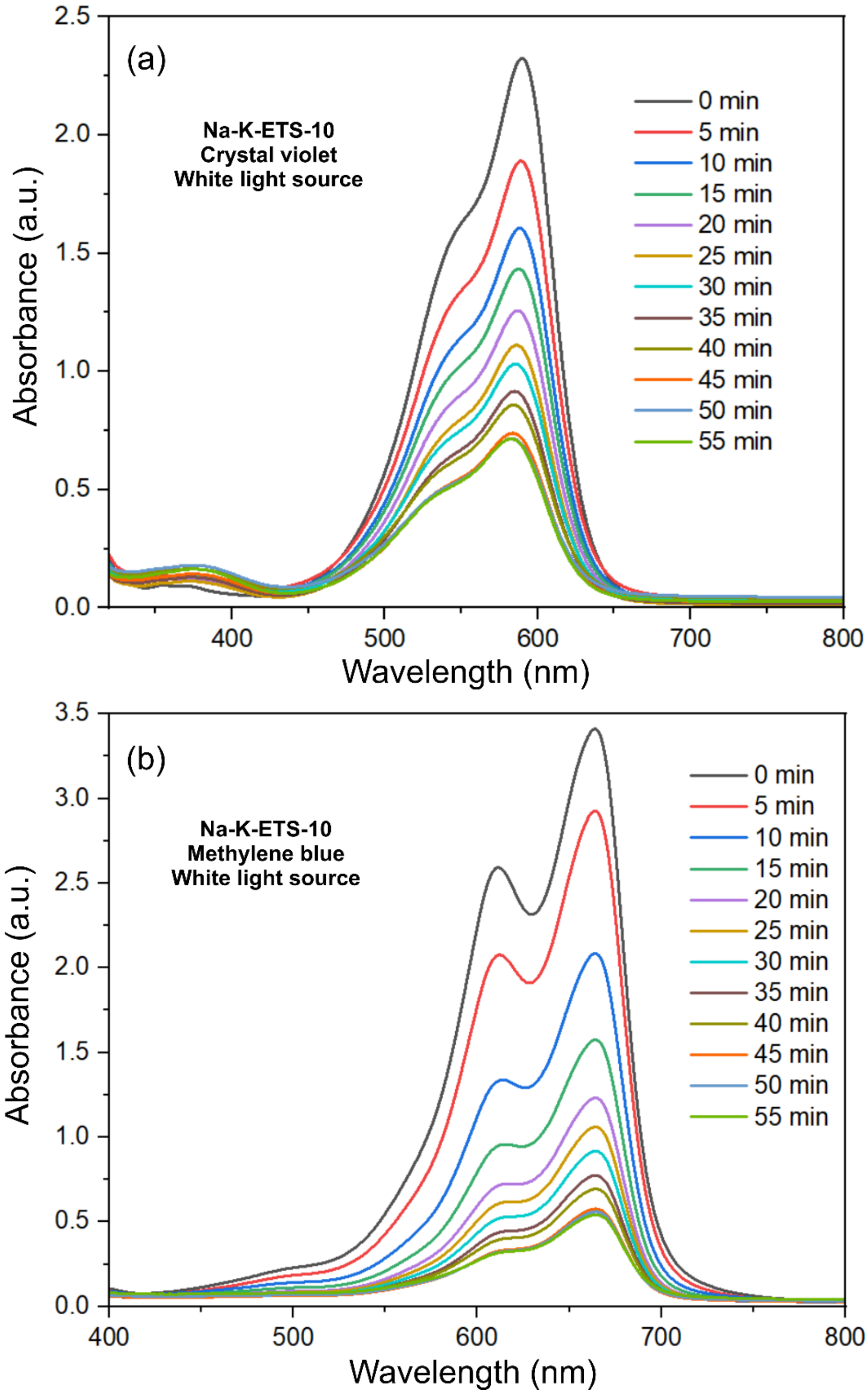

Figure S2. Photodegradation kinetics for Crystal violet (a) and Methylene blue (b) using Na-K-ETS-10 as catalyst under white light radiation.

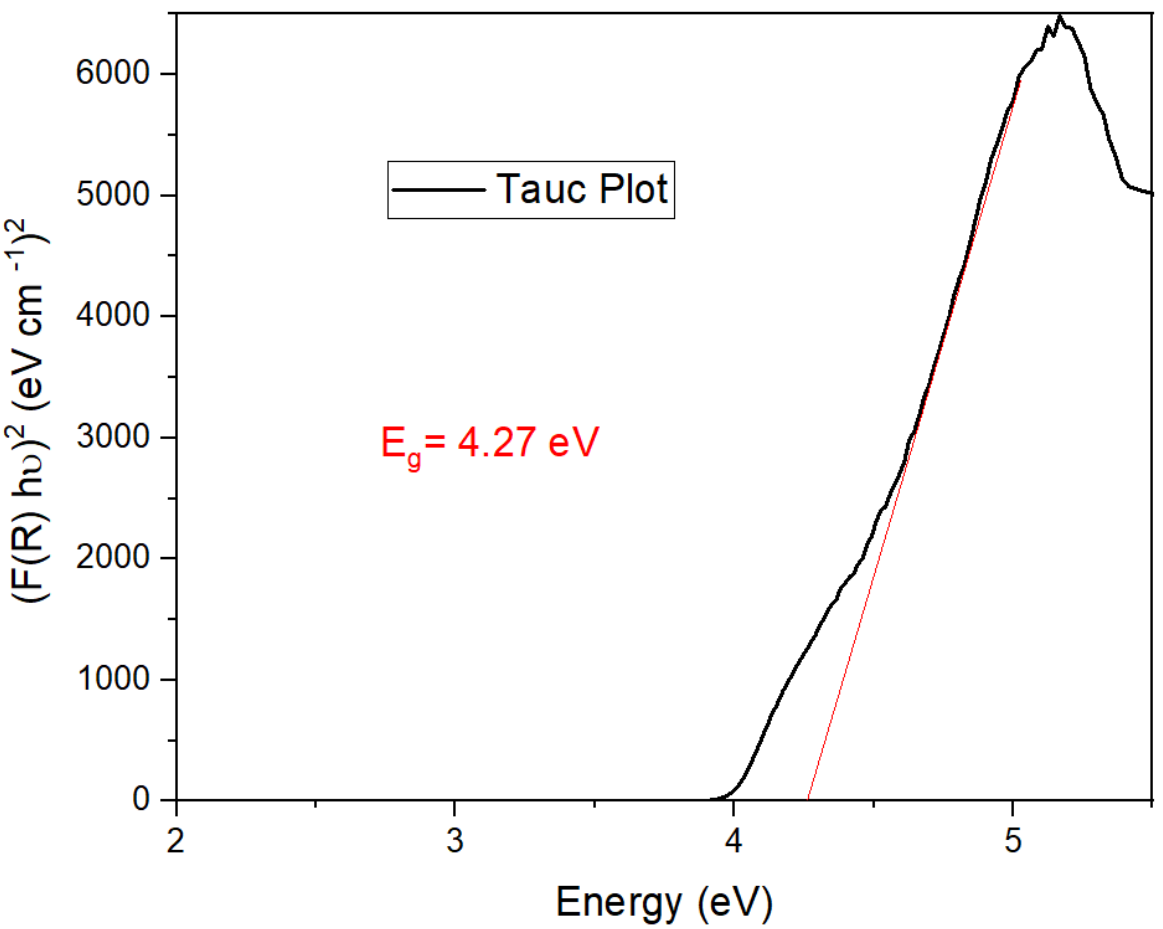

Figure S3. Tauc plot for Na-K-ETS-4/2.3Zr.

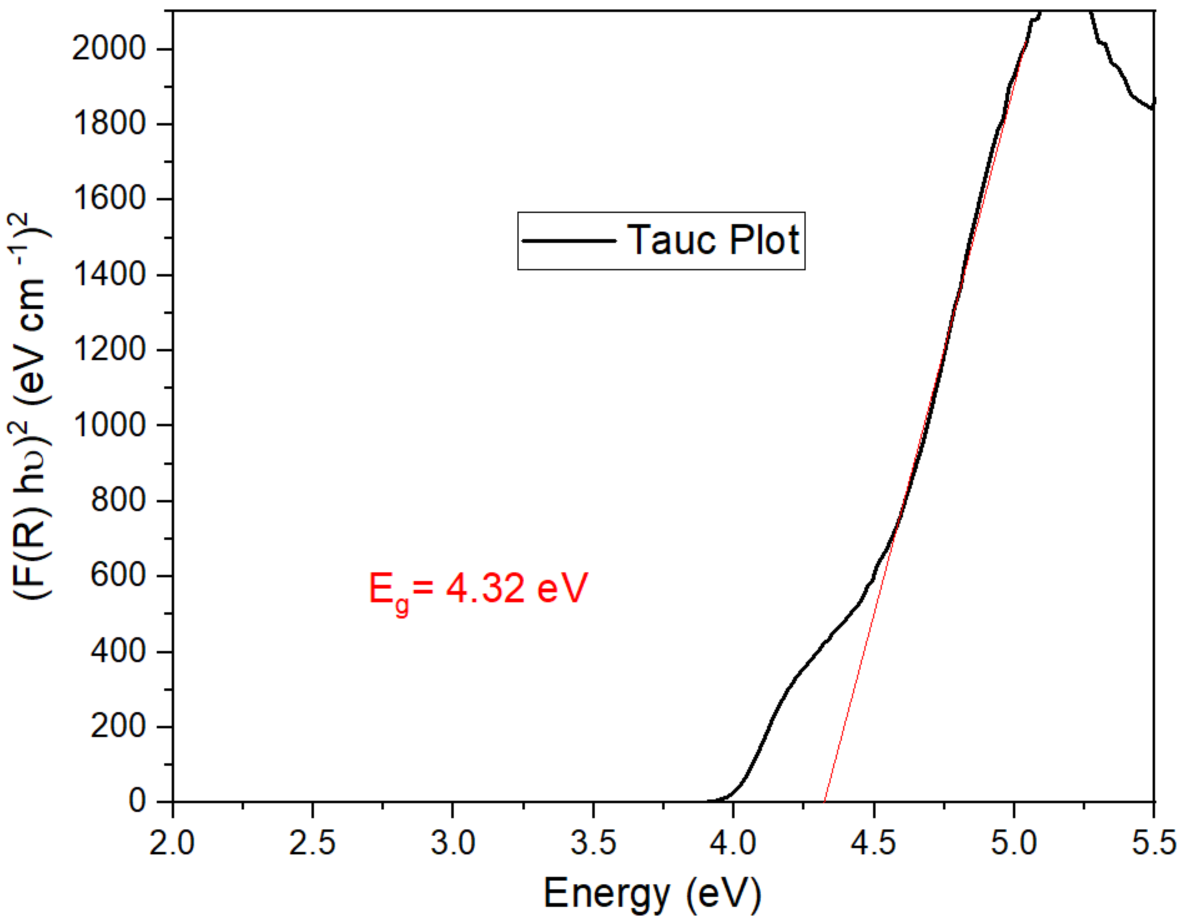

Figure S4. Tauc plot for Na-K-ETS-4/6.3Zr.

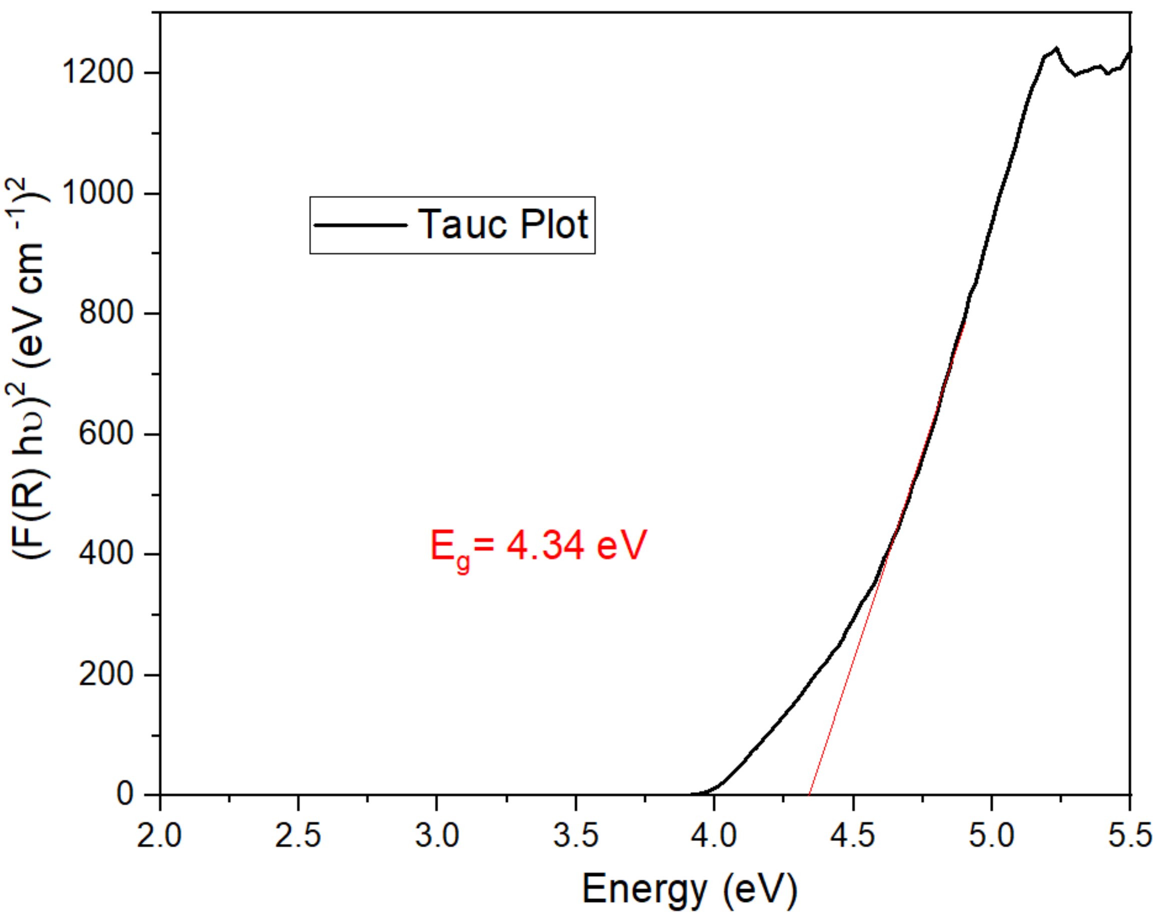

Figure S5. Tauc plot for Na-K-ETS-4/8.9Zr.

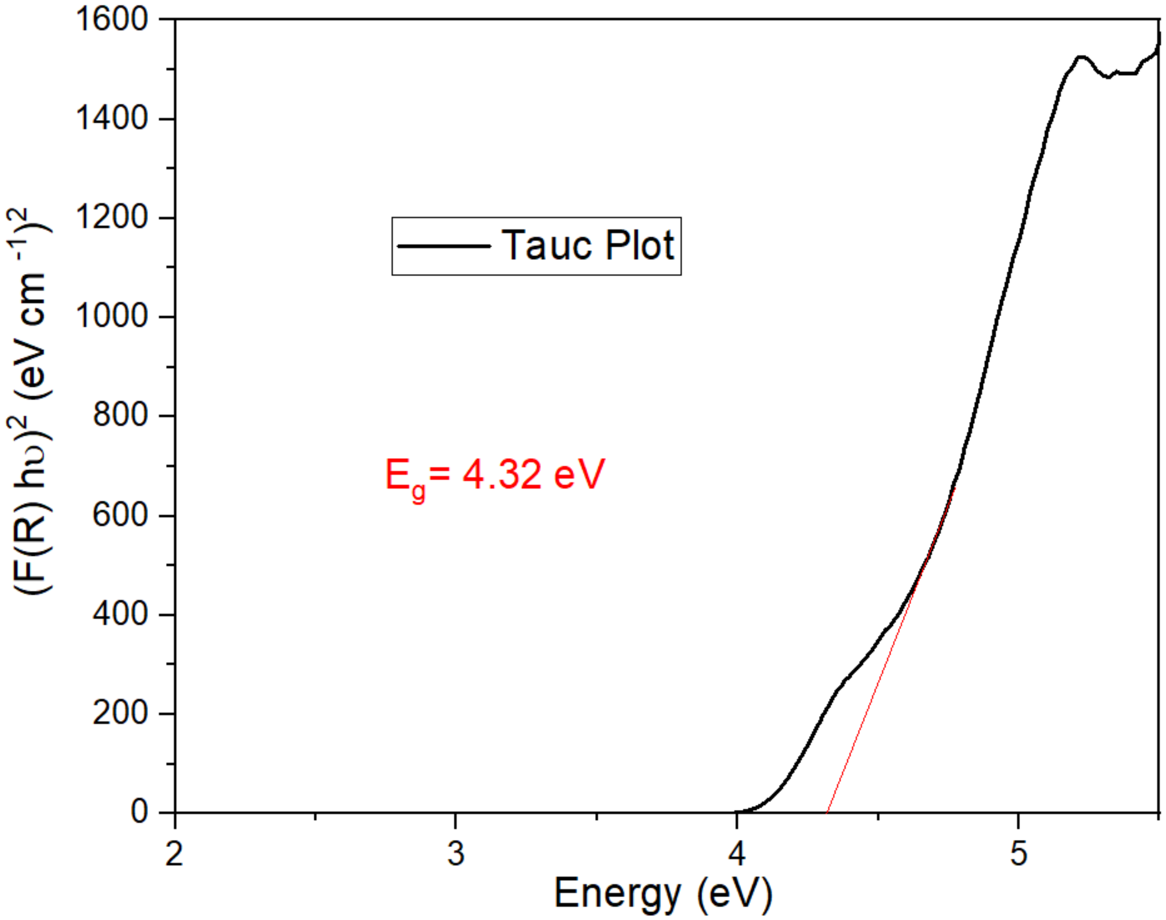

Figure S6. Tauc plot for Na-K-ETS-4/9.2Zr.

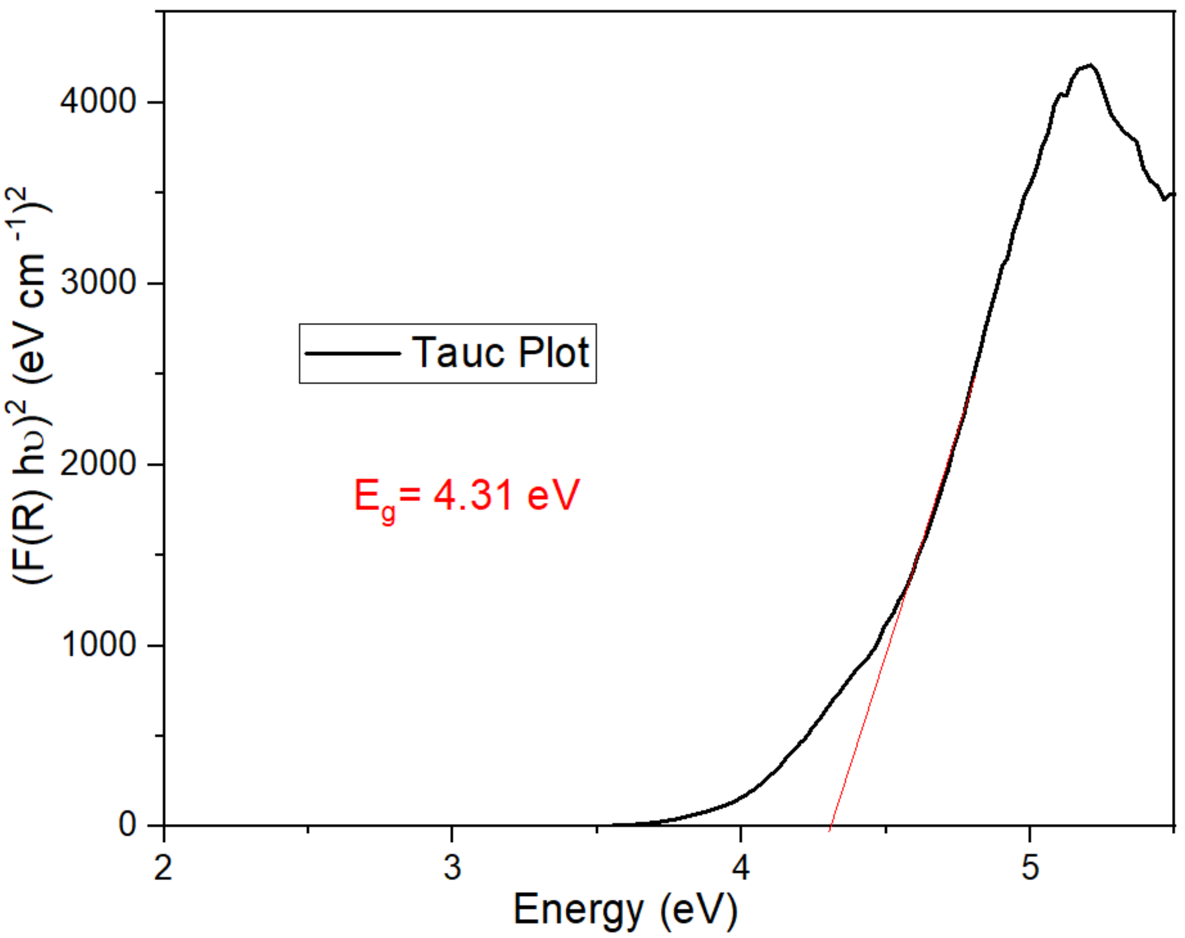

Figure S7. Tauc plot for Na-K-ETS-10/6.3Zr / Na-K-ETS-4/8.9Zr.

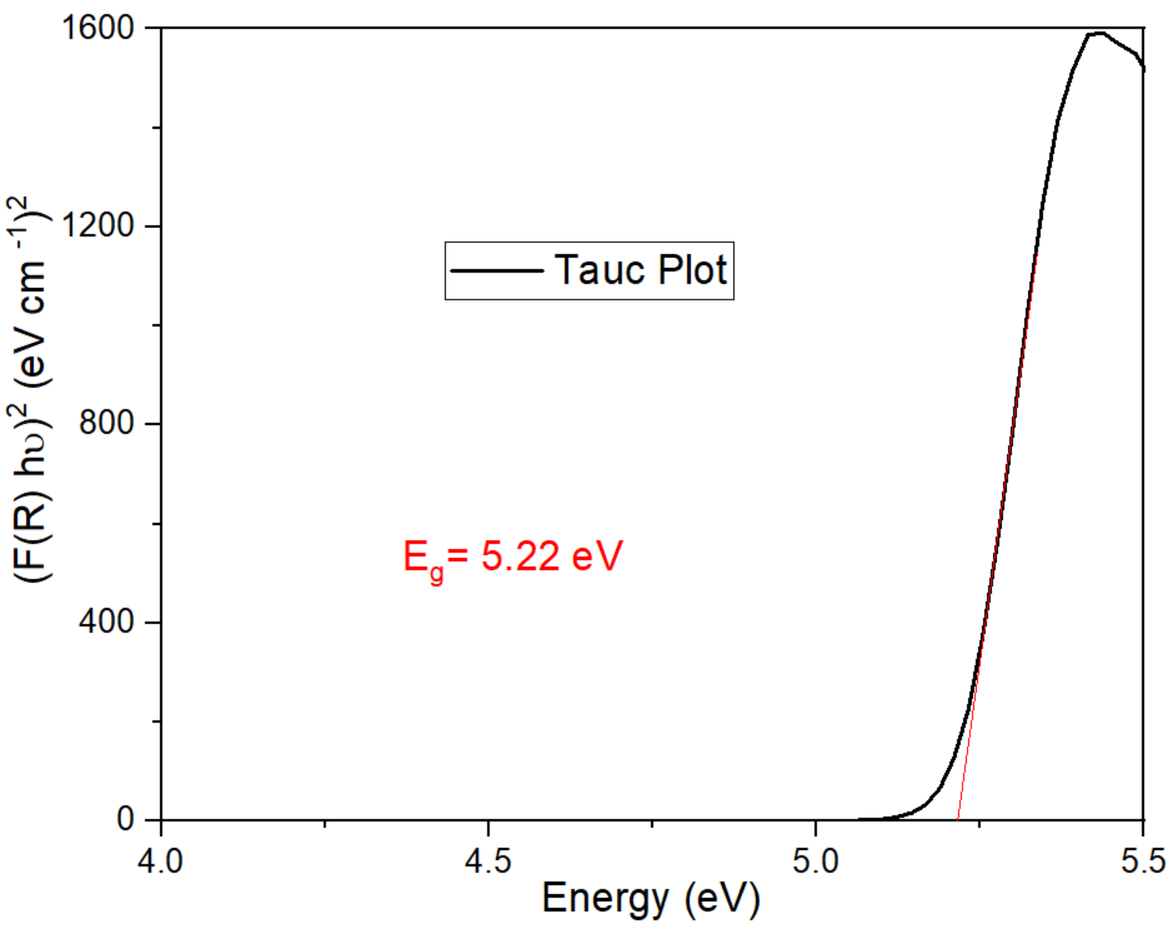

Figure S8. Tauc plot for ZrO_2.

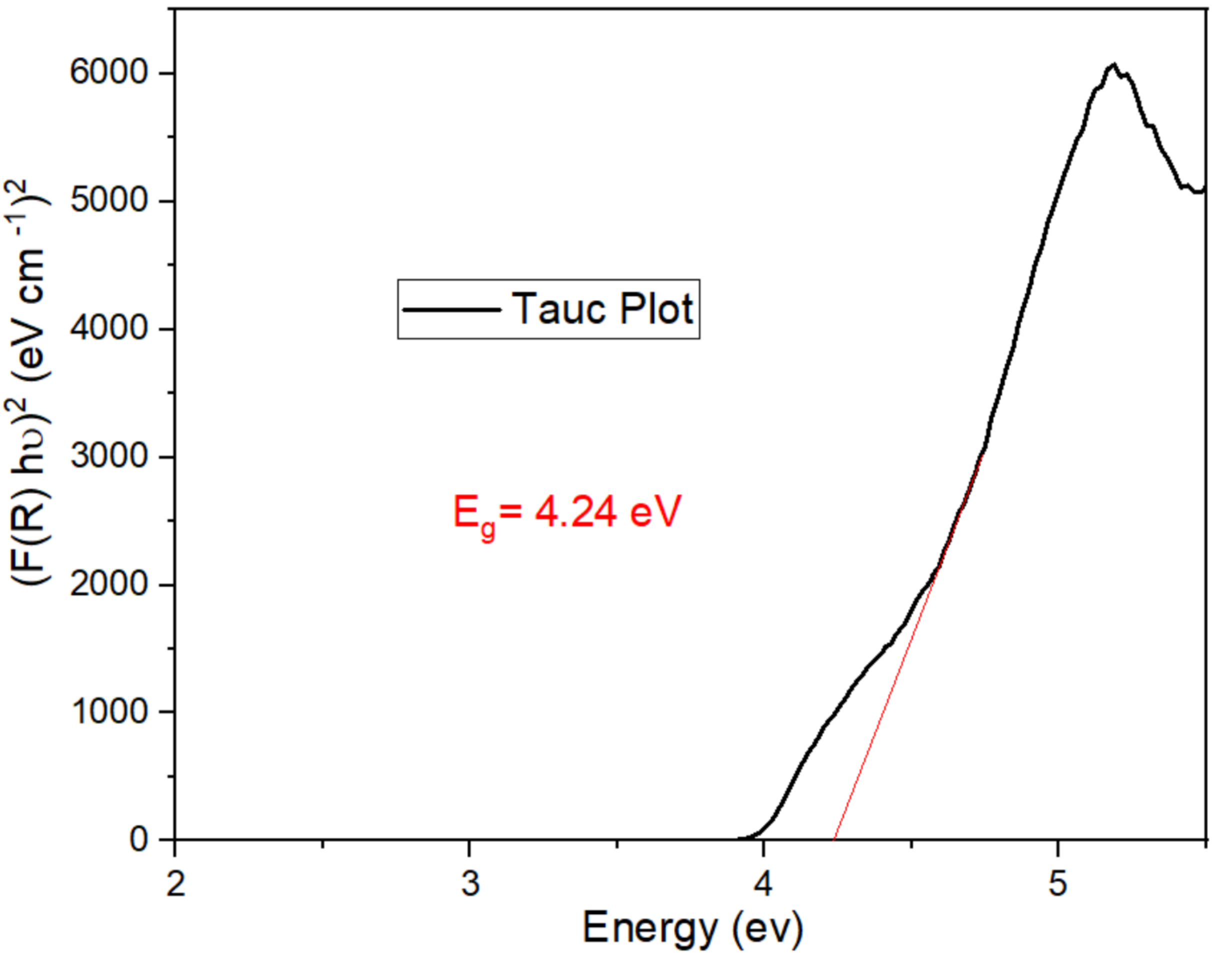

Figure S9. Tauc plot for Na-K-ETS-4.

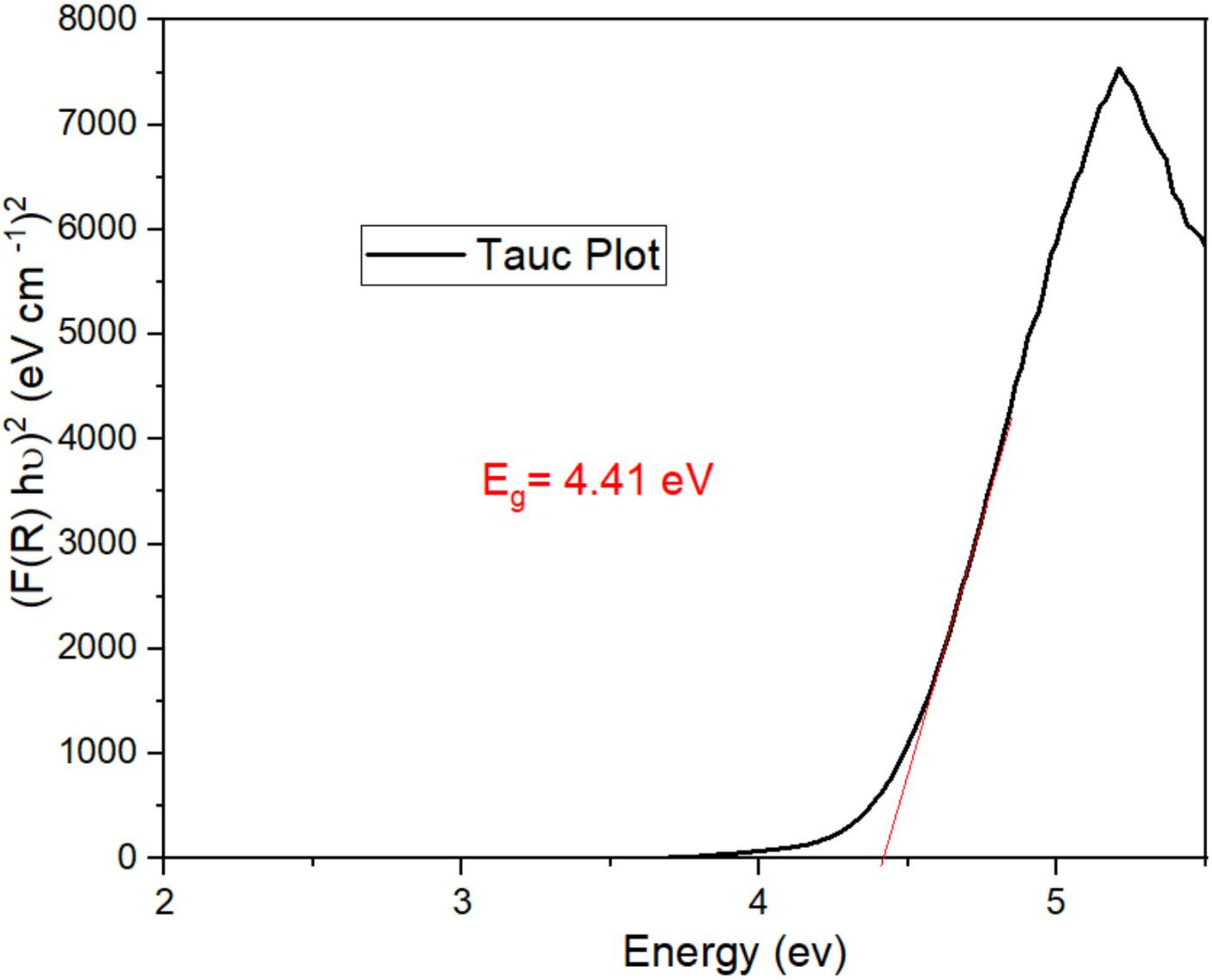

Figure S10. Tauc plot for Na-K-ETS-10.
